# Investigating the Impact of Positively Charged Gold Nanoparticle (AuNP) Concentration in Water/Cl^–^ Solutions Using Molecular Dynamics Simulations

**DOI:** 10.1021/acsomega.5c01441

**Published:** 2025-05-14

**Authors:** Esequias Coelho, Douglas Xavier de Andrade, Agnaldo Rosa de Almeida, Guilherme Colherinhas

**Affiliations:** † Instituto de Física, Universidade Federal de Goiás, 74690-900 Goiânia, Goiás, Brazil; ‡ Instituto Federal de Educação, Ciência e Tecnologia de Goiás, 74968-755 Aparecida de Goiânia, Goiás, Brazil; § Campus Anápolis de Ciências Exatas e Tecnológicas, Universidade Estadual de Goiás, 75132-400 Anápolis, Goiás, Brazil

## Abstract

This study presents a detailed analysis of the interactions between positively charged gold nanoparticles Au_144_(SRNH_3_^+^)_60_ and chloride ions (Cl^–^) in aqueous solution, using molecular dynamics simulations. Four systems with varying amounts of chloride ions were investigated: 60 Cl^–^, 120 Cl^–^, 180 Cl^–^, and 240 Cl^–^, alongside varying quantities of nanoparticles. The focus of this research is to elucidate the energies involved, hydrogen bonding patterns, and radial distribution of ions around the gold nanoparticles, providing a fundamental basis for evaluating the potential applications of these systems in disease treatment. The results reveal significant differences in the Coulomb and van der Waals interaction energies between nanoparticles and ions, as well as between nanoparticles and water molecules. Furthermore, this study highlights the patterns and lifetimes of hydrogen bonds between nanoparticles and water molecules, along with the mobility of system components in solution. These findings have important implications for potential applications in bionanotechnology, offering a deeper understanding of the interactions between ions and gold-based nanoparticles.

## Graphical abstract

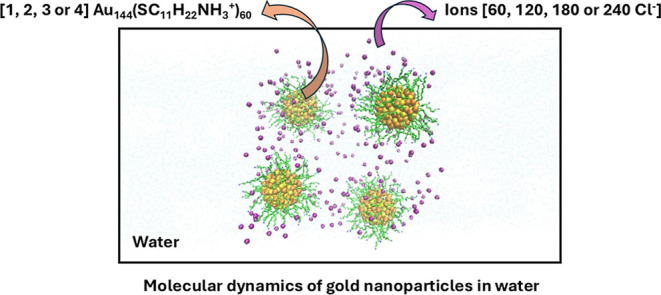

## 1 Introduction

Nanotechnology is present in several areas of knowledge, including physics, chemistry, biology and engineering,^1^ offering numerous applications and potential benefits in the treatment of diseases, such as cancer, and in improvements to imaging and diagnostic equipment.^2–5^ Beyond its impact on medicine, nanotechnology has also been widely explored in the cosmetic sector.^6,7^ Gold nanoparticles (AuNP) are known for their antibacterial and antifungal properties^8^ and are commonly found in products such as antiperspirants, antiaging creams and facial masks. Antimicrobial and rejuvenating effects are well documented and play a crucial role in both for the cosmeceutical industry and for wound healing treatments.^9^

Nanoparticles have been widely used in nanomedicine and biochemistry, especially for controlled drug delivery, diagnostics, therapies, bioimaging and in the detection of chemical and biological agents.^10–15^ One of the key advancements in this field has been the development of bioanalytical sensors, which exploit surface interactions to enable selective biorecognition reactions.^16^ When nanoparticles interact with the biological environment, molecular competition for adsorption on their surface occurs^17^ paving the way for the development of multifunctional diagnostics and innovative treatments for diseases.^18^ In this framework, AuNPs stand out as important nanoagents, with varied applications offering unique properties such as high conductivity and colorimetric sensitivity, which enhance their effectiveness in detecting biological molecules and related applications.

In medicine, AuNPs have demonstrated great potential as carriers for controlled drug, gene, and protein delivery, as well as in therapies such as photothermal therapy, photodynamic therapy, and radiotherapy, reinforcing their role in diagnostics and medical imaging.^19–21^ Among nanoparticles, AuNPs stand out due to their excellent biocompatibility, which, combined with structural stability and ease of functionalization with biologically active molecules, allows direct interactions with proteins, drugs, antibodies, enzymes, and nucleic acids.^22–24^ Particularly noteworthy are ultrasmall AuNPs, with diameters below 2 nm, which exhibit molecular definition and atomic monodispersity.^25^ These characteristics ensure consistent and predictable physicochemical properties, including stability, reactivity, and interactions with other molecules or surfaces. Such properties are especially relevant in materials science, biomedicine, and catalysis, where nanoparticle uniformity can directly influence system performance. However, despite the broad application of AuNPs, a detailed investigation of their interactions in aqueous solutions, particularly concerning their charge states in the presence of ions, remains underexplored in the literature. However, despite the wide application of AuNPs, a detailed investigation of the interactions, focusing on how they can be positively or negatively charged in the presence of ions in aqueous solutions is underexplored in the literature.

AuNPs are characterized by formulas as (Au)_*N*_(SR)_*M*_, where *N* e *M* represent combinations such as (12, 18), (38, 24) and (144, 60). These particles are composed of a specific number of gold atoms stabilized by thiolate ligands, which prevent aggregation and control particle size, differentiating them from larger metallic or plasmonic nanoparticles with cores exceeding 2 nm in diameter. Recently, ligand properties have been fine-tuned to optimize the functionality of AuNP surfaces for specific applications. Organic ligands, particularly thiolates, are widely used due to the strong affinity between gold and sulfur, denoted by the term SR.^26–30^ Currently, thiolates-protected AuNPs species have been widely applied in several areas, such as biology,^31,32^ catalyst design^33,34^ and chemiresistive sensors.^35^

Although AuNPs are employed in real biological environments, simulating their interactions within complex biological systems in aqueous media is computationally challenging. Therefore, approximation methods have provided valuable insights. For instance, simulations of AuNPs in aqueous solutions and their interactions with membranes have shed light on their behavior in biologically relevant conditions. In this context, molecular dynamics (MD) is widely recognized as a precise and reliable approach for studying nanomaterials in biological environments,^36–44^ allowing the determination of structural and energetic properties, direct interactions with cell membranes, and aggregation behavior.^45–48^ Heikkilä et al. highlight the importance of long-range electrostatic interactions in determining the properties of nanoparticles in aqueous solutions, as water mediates these interactions, which play a fundamental role in their interaction with cellular lipid membranes.^45^ The same study emphasizes that the nature of AuNP-membrane interactions depends on the nanoparticle charge and the functional groups present on their surface.^46^ Compared to the work of Heikkilä et al.,^45,46^ which focused on anionic AuNPs and their interactions with lipid membranes, our study provides new insights by examining cationic AuNPs in a chloride-rich aqueous medium. While both studies highlight the role of long-range electrostatic forces, our analysis further explores how nanoparticle concentration affects energetic balance and hydrogen bond dynamics, an aspect not addressed in previous work. Recent work such as that of Bordoni and Colherinhas et al. performed MD simulations to investigate the effects of functionalized AuNPs, specifically $Au_{144}{\left(SC_{11}H_{22}COO^{-}\right)}_{60}$ , at different concentrations in aqueous solutions with sodium ions. The results indicated that as the concentration of AuNPs increases, the formation of nanoparticle clusters occurs due to direct interaction with water molecules.^47^ Our findings reveal a partially similar effect, in which higher concentrations of positively charged AuNPs reduce their interaction with water and increase their interaction with chloride ions, indicating a tendency toward ion-mediated stabilization rather than simple aggregation. Reports in the literature indicate that negatively charged AuNPs in aqueous environments with different ionic compositions show that divalent ions (Mg^2+^, Ca^2+^) interact more significantly than with monovalent ions (Na^+^, K^+^) affecting mobility and hydration.^48^ Our study, which focuses exclusively on Cl^–^ ions, suggests that even monovalent ions can significantly alter the behavior of AuNPs, particularly in terms of Coulombic interaction strength and solvation effects.

In this study, we conduct classical MD simulations of positively charged AuNPs in aqueous environments with chloride ions, considering four systems at different AuNP concentrations. By analyzing hydrogen bond (HB) dynamics between AuNPs and water molecules, nanoparticle mobility, the spatial arrangement of water molecules and ions around AuNPs, and energetic interactions (Coulomb and Lennard-Jones), we aim to elucidate how nanoparticle concentration influences system behavior, providing insights for future AuNP applications.

## 2 Methodology

This work studies the behavior of the interactions between a positively charged AuNP of the Au_144_(SRNH_3_^+^)_60_ type and chlorine ions Cl^–^ with variations in the molar concentrations of AuNP in water solution using classical MD simulations. Table 1 shows the composition of the studied systems characterizing the different concentrations of AuNP in solution. To create the molecular structure of (Au)_144_(SRNH_3_^+^)_60_, displayed in Figure 1, the united atom concept was adopted, describing each -CH_2_- group as a single particle during the computational simulation. The carbon chain –R– composed by C_11_H_22_ has a polar head containing an amine group (–NH_3_^+^), connected to the gold core (Au_144_) of the AuNP through a sulfur atom (S).^45^ Each AuNP has a total charge of −60*e*, resulting from the functionalization with 60 amine groups (–SRNH_3_^+^). Au_144_(SRNH_3_^+^)_60_ structure followed the procedure described by Heikkilä et al.^45^ and the proposed force field combines OPLS-AA parameters^49^ to describe each component of the AuNP structure in solution. It is important to note that the gold core (Au_144_) provides structural stability and essential electronic properties, while interacting minimally with the surrounding medium due to its low chemical reactivity. In contrast, the functionalized region ((SRNH_3_^+^)_60_), composed of thiolated ligands with positively charged terminal amine groups, plays a central role in electrostatic interactions and in stabilizing the solvation layer, thereby governing the dispersion and behavior of the nanoparticles in chloride-rich aqueous solutions. In this study, the Au_144_ cluster adopts a rhombicosidodecahedral geometry which is characteristic of this specific type of nanoparticle. The modeling approach and force field parameters used here are based on the structure described by Heikkilä et al.,^45^ ensuring that the rhombicosidodecahedral geometry is preserved throughout the simulations. The gold core maintains this geometry, while the thiolate ligands (SRNH_3_^+^) form a functional shell around the metallic core, which is essential for stabilizing the nanoparticle in aqueous solution and mediating interactions with ions and water molecules. This geometric stability was taken into account during the MD simulations, and no significant structural deviations from the original rhombicosidodecahedral shape were observed. Therefore, the geometry described in the reference was maintained in the modeled nanoparticles, ensuring consistency with previous studies. Water molecules were modeled using the Simple Point Charge model (SPC).^50^ Simulations were performed with the Gromacs program (Version 2022)^51^ and figures were produced using the VMD program (Version 1.9.4).^52^

**Table 1:** Composition of Simulation Boxes Containing AuNP, Ions and Water Molecules^a^

system	# AuNP	# Cl^–^	# water	initial volume	final volume	molar concentration (10^–5^ mol/mL)
configuration-01	1	60	3351	125.0	126.71 ± 0.54	1.31
configuration-02	2	120	5632	216.0	220.73 ± 0.69	1.51
configuration-03	3	180	9134	343.0	352.16 ± 0.84	1.41
configuration-04	4	240	14,152	512.0	529.67 ± 1.07	1.25

a Initial volume corresponds to the exact volume (in nm^3^) of the configuration generated with the Packmol program and the final volume corresponds to the average of the volume obtained in the last 100 ns MD-simulation (in nm^3^ ±RMSD), along with the molar concentration of AuNPs in water (in mols/mL).

**Figure 1:**
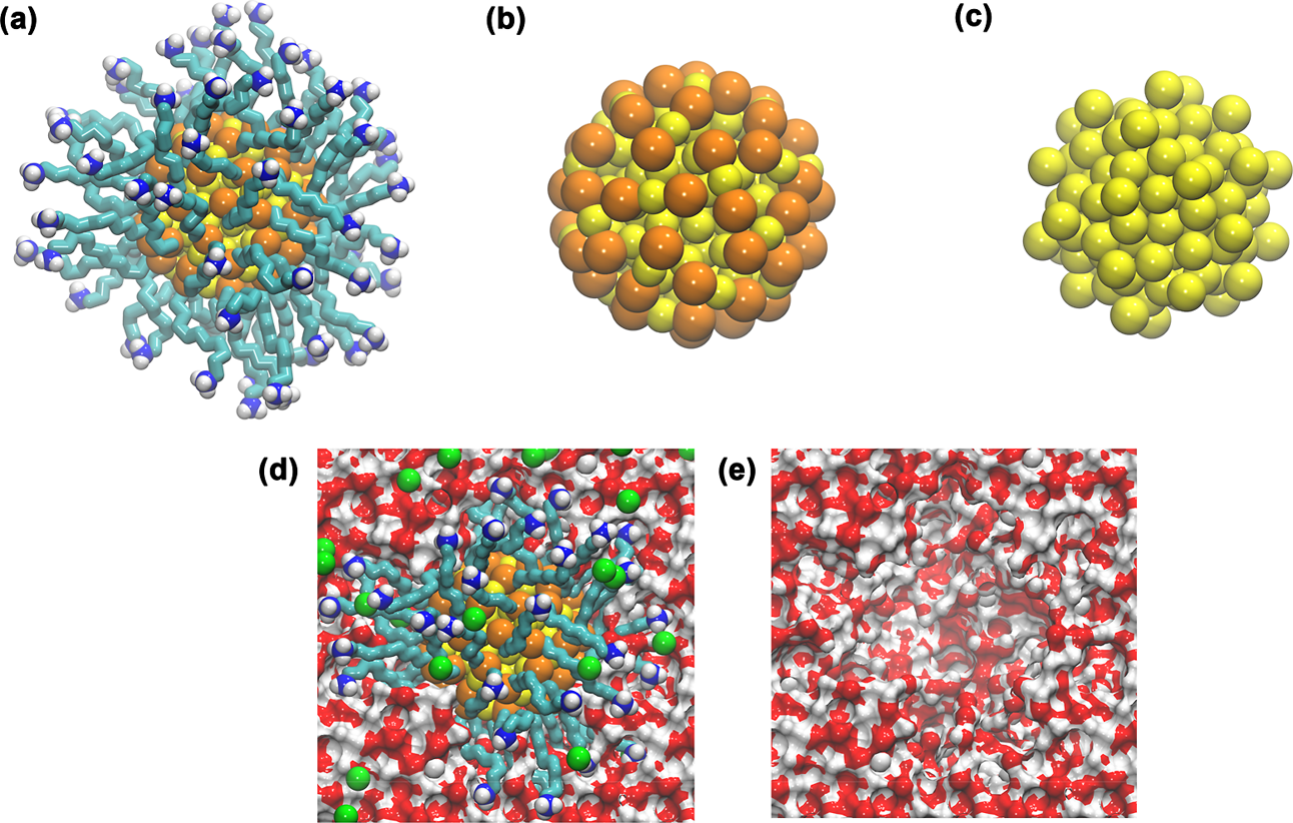
(a) AuNP–Au_144_(SRNH_3_^+^)_60_; (b) S and Au atoms; (c) only Au atoms; (d) AuNP and ions in water cavity; and (e) only water cavity. The –R– carbon chain is composed of R = C_11_H_22_ (cyan) and has a polar head containing an amine group (–NH_3_^+^) (blue and white). Connected to the gold core (Au_144_) of the AuNP (yellow) through a sulfur (S) atom (orange).

Initially, AuNPs and ions were randomly distributed in a simulation box using the Packmol program,^53^ forming four different configurations consisting of: a single AuNP solvated with 60 chloride ions (60 Cl^–^) in 125.0 nm^3^ (configuration-01); two AuNPs solvated with 120 chloride ions (120 Cl^–^) in 216.0 nm^3^ (configuration-02); three AuNPs solvated with 180 chloride ions (180 Cl^–^) in 343.0 nm^3^ (configuration-03); and four AuNPs solvated with chloride ions (240 Cl^–^) in 512.0 nm^3^ (configuration-04), all empty spaces of the simulation boxes were filled with water molecules. Table 1 shows the composition of each system, the number of simulated atoms, the initial volume and the average of the final volume and the molar concentration of AuNPs in aqueous solution, in mols/mL. For each configuration, we performed MD simulations alternating between the *NVT* and *NPT* ensembles for 50 ns. After the system reached thermodynamic equilibrium, all configurations were subjected to a production stage with simulations in the *NPT* ensemble for 100 ns. Every 0.001 fs, the particle positions and velocities were updated, maintaining the temperature at 300 K using v-rescale algorithm^54^ at constant pressure (1.013 bar) by using isotropic Parrinello–Rahman coupling,^55^ generating 10^8^ configurations, of which 5 × 10^4^ configurations were saved for statistical analysis. LINCS algorithm was used to maintain the integrity of intramolecular bonds.^56^ To ensure the validity of the production trajectory, the systems were evaluated in terms of equilibrium and convergence criteria. The choice of a 100 ns simulation time was based on the observation that the system had reached thermodynamic equilibrium, as evidenced by the stability of key observables throughout the trajectory. Parameters such as interaction energies (Coulomb and Lennard-Jones), radial distribution functions (RDF), mean square displacement (MSD), and hydrogen bond (HB) numbers fluctuated around stable average values without showing considerable drift over time. This indicates that the sampled configurations are representative of a statistically converged and equilibrated ensemble (RMSD graphs for all systems are presented in the support material). This protocol has demonstrated excellent results in solvated organic systems as can be seen in the refs 44,55,57–60 . In order to investigate the properties of HBs, such as lifetime and free energy for rupture, we adopted the methodology proposed by Luzar, Chandler and van der Spoel.^61–63^ This approach allows the analysis of HBs dynamics through the correlation function, which quantifies the probability of a bond remaining intact throughout the simulation time, providing information about the correlation between the existence of a bond at different times. From these analyses, it was possible to estimate the Gibbs free energy associated with the formation of HBs, considering the spatial and angular distribution of the molecules as *r* ≤ 3.5 nm (distance between the two donor–acceptor atoms) and θ ≤ 30*°* (acceptor-H-donor angle).

## 3 Results and Discussion

### 3.1 Interaction Energy

The averages of the Coulomb interaction energies (*E*_C_) and Lennard-Jones (*E*_LJ_), in kJ/mol, were analyzed between peers AuNP–AuNP, AuNPs–ions and AuNPs–water, for the four configurations studied in this work (as shown in Tables 2–5). It can be observed that the AuNP–AuNP electrical self-interaction presented results with small variations (Table 2), with average *E*_C_ ranging from −11,529.5 to −11,300.1 kJ/mol, which represents a maximum variation of ∼2%. Thus, regardless of the amount of ion used in the wet solution, the AuNP maintains a high electrical self-interaction energy, as expected. However, the presence of other AuNPs in the simulation may be responsible for this decrease. As can also be seen, the electrical interactions between the AuNPs in solution can be considered practically null compared to the intramolecular values. Results for *E*_C_ between different AuNPs do not exceed 4 kJ/mol. Table 3 shows the average electrical interactions between the AuNPs-chlorine ions and AuNPs-water molecules. We found that increasing the amount of AuNPs results in a reduction of ∼3% in the amount of *E*_C_ (AuNPs-water). This decrease can be explained by the change in the molar concentration of AuNPs in aqueous solution, which at higher molar concentrations results in competition for water molecules by the AuNPs, leading to a reduction in the number of water molecules directly interacting with the AuNP, resulting in the observed decrease in the interaction energy. Despite the slight decrease in the interaction between the AuNPs and the water molecules, we observed a percentage variation in the Coulomb energy *E*_C_ between ∼8% (configuration-03) and −2% (configuration-04) compared to configuration-01 in the interaction between AuNPs and ions. This intensification of the ionic interaction can be explained by the higher molar concentration of AuNPs in configuration-03, which favors the capture of ions by the nanoparticles, unlike what happens in configuration-04, where there is a decrease in the molar concentration and consequently a lower capture of ions by the AuNP.

**Table 2:** Average Coulombic (*E*_C_) Interaction Energies for Interaction of Individual AuNPs (in kJ/mol) for Configurations-1 to 4^a^

system	configuration-01	configuration-02	configuration-03	configuration-04
AuNP #1–AuNP #1	–11,529.5 ± 1.9	–11,301.4 ± 1.6	–11,301.4 ± 1.4	–11,300.8 ± 1.0
AuNP #1–AuNP #2		0.8 ± 0.1	3.3 ± 0.7	0.2 ± 0.1
AuNP #1–AuNP #3			2.6 ± 0.4	0.03 ± 0.02
AuNP #1–AuNP #4				0.16 ± 0.06
AuNP #2-AuNP #2		–11,300.8 ± 1.5	–11,299.0 ± 1.4	–11,303.1 ± 1.4
AuNP #2–AuNP #3			2.4 ± 0.3	0.4 ± 0.1
AuNP #2–AuNP #4				0.05 ± 0.02
AuNP #3-AuNP #3			–11,299.8 ± 0.9	–11,302.0 ± 2.6
AuNP #3–AuNP #4				0.09 ± 0.03
AuNP #4–AuNP #4				–11,302.1 ± 1.2

a RMSD are shown for all average interaction.

**Table 3:** Average Coulombic (*E*_C_) Interaction Energies AuNPs–Ions and AuNPs–Water Molecules (in kJ/mol) for Configurations-1 to 4 per Number of the AuNPs in Solution^a^

system	configuration-01	configuration-02	configuration-03	configuration-04
AuNP–ions	–1270.4 ± 2.7	–1313.3 ± 5.3	–1377.2 ± 12.7	–1242.3 ± 5.8
AuNP–water	–15,208.5 ± 6.3	–14,826.2 ± 7.6	–14,756.6 ± 12.8	–14,894.9 ± 7.3

a RMSD are shown for all average interaction.

The average values of the Lennard-Jones interaction energy (*E*_LJ_) for self-interaction AuNP–AuNP (Table 4) presented results with small variations that are between −3283.9 and −3245.8 kJ/mol which represents a maximum variation of ∼1%, as can be seen in Table 4. The results obtained for *E*_LJ_ does not differ from that observed for the Coulomb interaction energy. Table 5shows the *E*_LJ_ values between AuNPs-water molecules and AuNPs-solvated ion. For AuNPs-ion interactions, we observed a variation between ∼6% (configuration-03) and −2% (configuration-04) compared to configuration-01, like the Coulomb energy results. However, the average *E*_LJ_ values between AuNPs and water molecules indicate different interaction patterns between these particles since we observed a difference of up to 53 kJ/mol between configuration 1 and 2 (reduction of ∼17%), due to the increase in AuNPs competition for water molecules as we increase the concentration of nanoparticles in solution.

**Table 4:** Average Lennard-Jones (*E*_LJ_) Interaction Energies for Interaction of Individual AuNPs (in kJ/mol) for Configurations-1 to 4^a^

system	configuration-01	configuration-02	configuration-03	configuration-04
AuNP #1–AuNP #1	–3263.6 ± 5.8	–3259.4 ± 9.0	–3266.1 ± 3.5	–3245.8 ± 5.2
AuNP #1–AuNP #2		–0.04 ± 0.003	–0.2 ± 0.04	–0.01 ± 0.004
AuNP #1–AuNP #3			–0.1 ± 0.02	–0.001 ± 0.001
AuNP #1–AuNP #4				–0.01 ± 0.003
AuNP #2–AuNP #2		–3260.2 ± 6.2	–3283.9 ± 6.6	–3248.9 ± 6.3
AuNP #2–AuNP #3			–0.1 ± 0.02	–0.02 ± 0.006
AuNP #2–AuNP #4				–0.003 ± 0.001
AuNP #3–AuNP #3			–3265.7 ± 4.3	–3261.4 ± 4.9
AuNP #3–AuNP #4				–0.004 ± 0.001
AuNP #4–AuNP #4				–3264.8 ± 3.5

a RMSD are shown for all average interaction.

**Table 5:** Average Lennard-Jones (*E*_LJ_) Interaction Energies between AuNPs–Ions and AuNPs–Water Molecules (in kJ/mol) for Configurations-1 to 4 per Number of the AuNPs^a^

system	configuration-01	configuration-02	configuration-03	configuration-04
AuNP–ions	–69.2 ± 0.1	–70.3 ± 0.3	–73.0 ± 0.5	–67.5 ± 0.4
AuNP–water	313.7 ± 7.2	260.7 ± 8.0	281.9 ± 20.4	279.9 ± 6.6

a RMSD are shown for all average interaction.

It is important to emphasize that for the AuNP–AuNP pair, the average ratio between the Coulomb and Lennard-Jones interaction energies remains close to 3.5 for all analyzed systems, indicating an energy balance of the AuNP in solution. For the AuNP-ions pair, this ratio is ∼19 while for the AuNP-water pair the ratio is between ∼49 and 57, demonstrating how the Coulomb interaction energy significantly dominates the interaction. These results demonstrate that electrostatic forces are predominant in the interactions between AuNPs in solution and the similarity found for systems with different concentrations suggests that the system reaches a rapid energy convergence as the AuNP concentration increases. The high predominance of the Coulomb interaction energy in AuNP-water interactions, especially for configuration 1, can be attributed to the formation of a less competitive solvation layer among the other AuNPs that are present in the system in the other configurations. Furthermore, the presence of ions in the solution can influence the thickness and structure of the solvation layer, also affecting the intensity of Coulomb interactions between AuNPs and water molecules. This understanding is fundamental for systems composed of stable AuNPs, where the prevention of nanoparticle agglomeration can be crucial for their applications in biosensors and controlled drug delivery systems. The strong electrostatic interaction between AuNPs and chloride ions affects the hydration layer and colloidal stability, both of which are critical for drug delivery systems, where dispersion in physiological media prevents aggregation and loss of bioavailability. Moreover, the stability of HBs surrounding the functionalized amine groups (–NH_3_^+^) suggests an enhanced potential for drug transport via electrostatic interactions. In biosensors, for instance, the influence of AuNP concentration on ion distribution highlights the need for precise control of nanoparticle density to prevent undesired aggregation, thereby ensuring sensor sensitivity. The interactions with chloride ions may also be leveraged in the design of sensors for the detection of anionic species. For applications in photothermal therapy and biomedical imaging, the interactions with water molecules and ions suggest that modifications in AuNP functionalization could optimize dispersion and bioactivity, directly impacting the efficiency of heat transfer in photothermal treatments.

### 3.2 Hydrogen Bond Structure and Dynamics

Table 6 shows the average HBs (per AuNPs) formed between AuNPs and water molecules in solution. HBs are attractive intermolecular interactions that occur between regions of high electron density of molecules and may indicate the grouping of these molecules in the simulation box.^64^ The parameters used to obtain the average HBs were θ ≤ 30° and *r* ≤ 3.5 nm. Despite the variation in the molar concentration of AuNPs in solution, the average HBs values are ∼170 HBs per AuNP. This constant value suggests that in aqueous systems with different ionic concentrations, AuNPs could be used without drastically changing the dynamics of the first solvation layer, especially for water molecules that are in direct interaction with the AuNP functionalization region. This is relevant for applications that depend on stability in the dispersion of nanoparticles in aqueous media, such as in biological or catalytic systems. It can also be noted that the HBs formed between the AuNPs and the water molecules have an average lifetime of ∼15 ps for the four configurations. HBs also display a critical rupture energy (Gibbs free energy, Δ*G*) of ∼11 kJ/mol, which represents a considerably high value. This result indicates a strong hydration of the nanoparticles in the functionalized region of the AuNP, driven mainly by electrical interactions. These energetic interactions directly influence the formation and stability of the HBs, which is reflected in the average lifetime and Gibbs free energy of these interactions. These results also show that, despite the variation in the molar concentrations of the nanoparticles, the global behavior of the HBs and the energetic stability are practically unchanged.

**Table 6:** Number of HBs Established between AuNPs and Water Molecules^a^

system	configuration-01	configuration-02	configuration-03	configuration-04
#HBs				
AuNP #1	170.4	170.4	170.4	170.4
AuNP #2		170.4	170.3	170.4
AuNP #3			170.4	170.4
AuNP #4				170.4
Average	170.4	170.4	170.4	170.4
Lifetime [ps]				
AuNP #1	15.0	14.6	14.9	14.8
AuNP #2		14.6	15.3	14.8
AuNP #3			14.5	14.4
AuNP #4				14.8
Average	15.0	14.6	14.9	14.7
Δ*G* [kJ/mol]				
AuNP #1	11.2	11.2	11.2	11.2
AuNP #2		11.2	11.3	11.2
AuNP #3			11.2	11.1
AuNP #4				11.2
Average	11.2	11.2	11.2	11.2

a Forward HB-lifetime of these interactions is also highlighted (in ps). Parameters used to obtain the average HB: θ ≤ 30° and *r* ≤ 3.5 nm. The forward HB-lifetime and Δ*G* for breaking the HB interactions was obtained using autocorrelation according to the theory of refs 61–63 .

### 3.3 Mass Density Profile

In this section, we will present the mass density profile for each component for all systems, revealing the structural characteristics of the molecules and particles inside the simulation box. In Figures 2, 3 and 4, the mass density profiles are presented along *x*, *y* and *z* axes, respectively. For a better understanding, the mass density profiles are centered with respect to AuNP #1 (black curve) which is in the center of the simulation box. In Figure 2, we present the mass density profile, *x* axis, for AuNPs, ions and water structures. In Figure 2a, for configuration-01, the mass density profile of the water molecules (red line) presents a slight reduction in the central region around the nanoparticle and the ions have an almost uniform distribution along the simulation box (magenta line). In Figure 2b, for configuration-02, we see the mass density profile of the two AuNPs (black and green lines) creating two regions with a slight decrease in water density where it is possible to see a slight increase in the mass density of water molecules between AuNPs. A similar pattern is observed for the ion density profile, which shows its highest mass density intensity located between the positions of maximum mass density intensity of the AuNPs. (peak near *x* = 1.0 nm and *x* = 3.5 nm in the simulation box). In Figure 2c, configuration-03, three AuNPs (black, green, blue lines) assemble a complex distribution, forming two low-density zones in the mass density profile of the water molecules. In this configuration, the relative position of two AuNPs are overlapped, showing that the three AuNPs are in a triangular position within the simulation box, where AuNP #1 is centered and the others are at the box boundaries, approximately *x* = 2 nm away from AuNP #1. In Figure 2d, configuration-04, with four AuNPs (black, green, blue, yellow lines), there are multiple low-density zones in the mass density profile of water molecules, resulting in quasi-homogeneous distribution of ions in solution.

**Figure 2:**
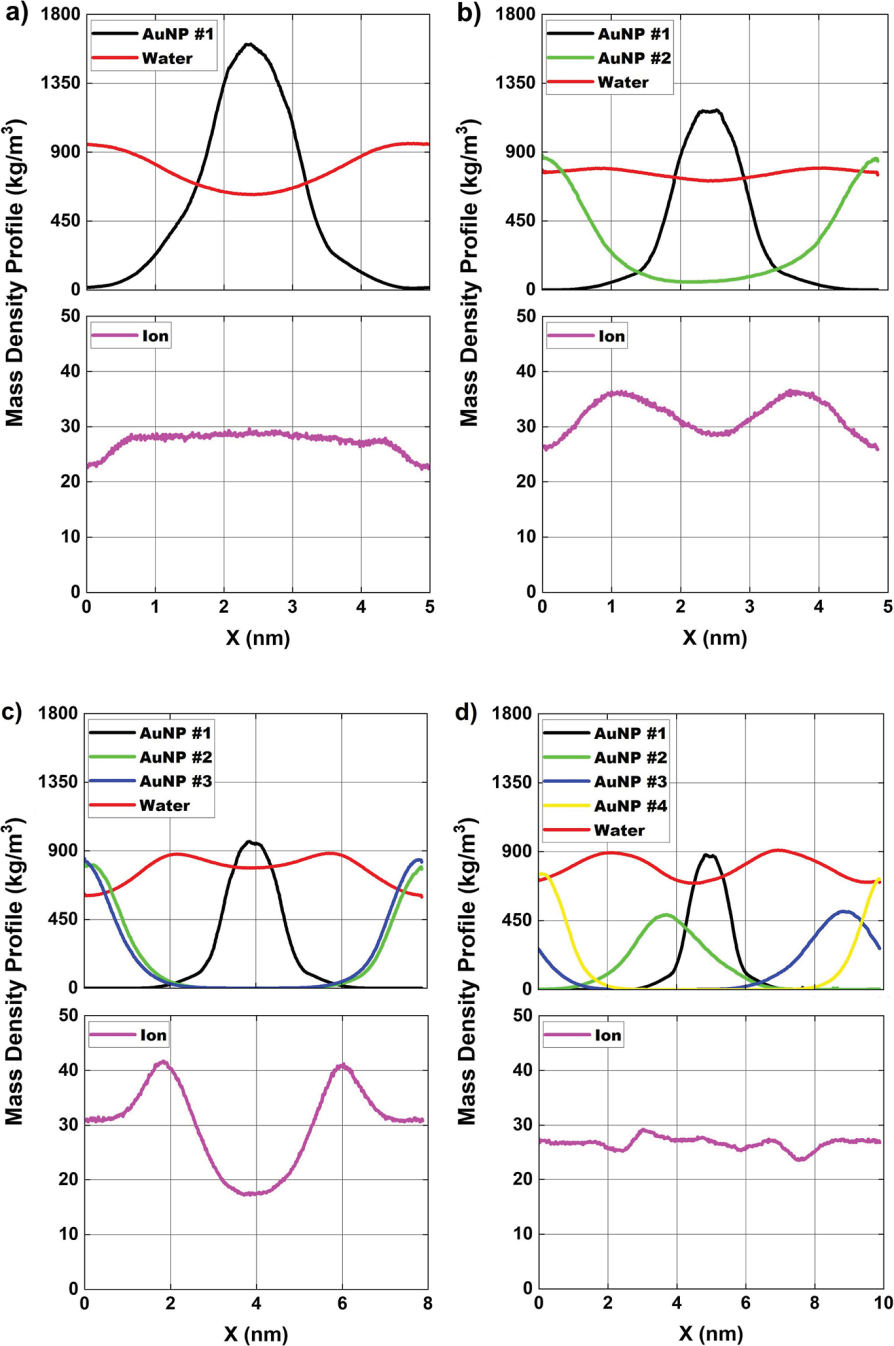
Average mass density profile [in kg/m^3^] of AuNPs and ions in solution and atomic mass for (a) configuration-01; (b) configuration-02; (c) configuration-03 and (d) configuration-04, along the *x*-axis of the simulation box. Magenta = Cl^–^ ions, black = centralized AuNP #1, AuNP #2 = green, AuNP #3 = blue, AuNP #4 = yellow and red = water.

**Figure 3:**
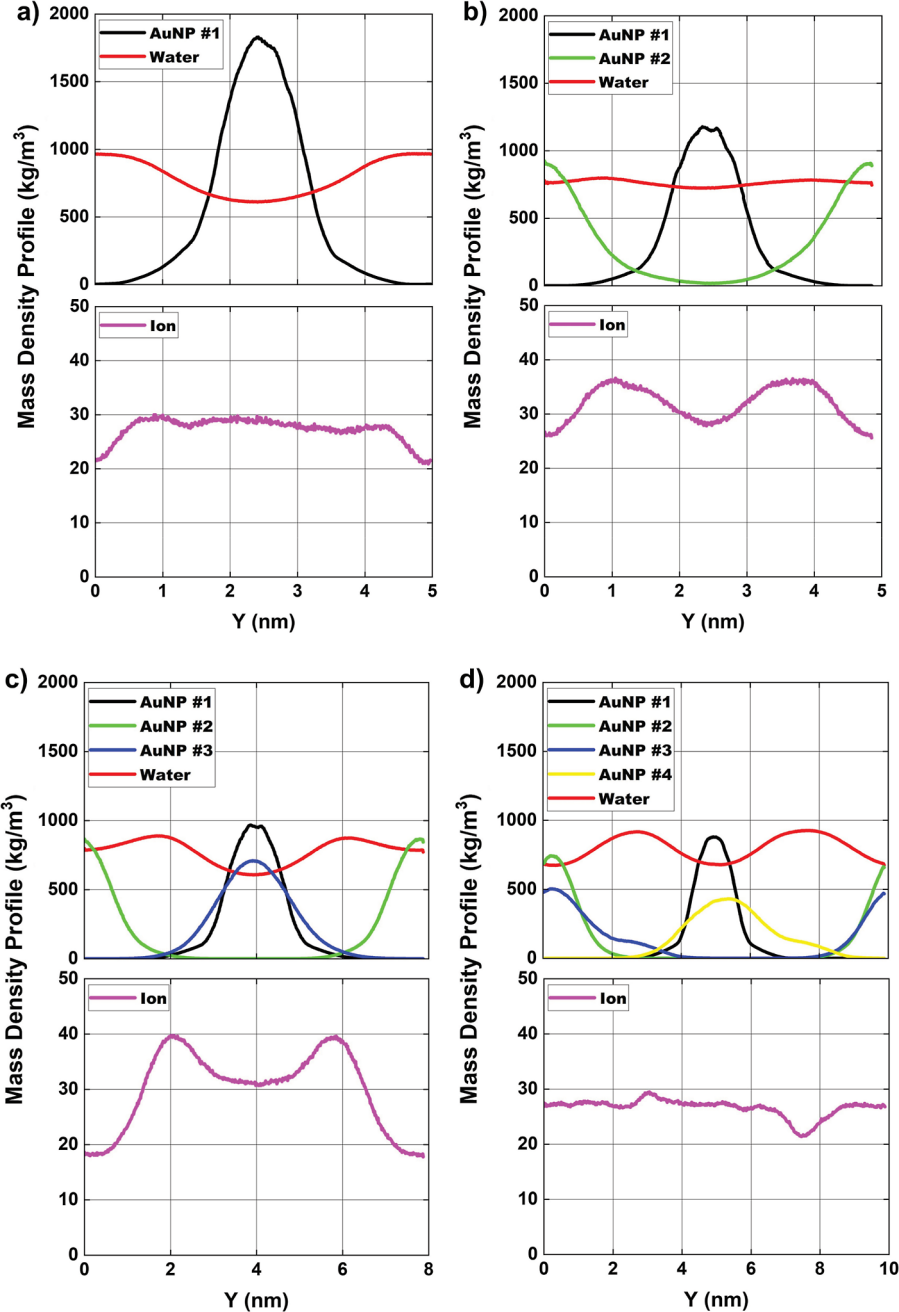
Average mass density profile [in kg/m^3^] of AuNPs and ions in solution and atomic mass for (a) configuration-01; (b) configuration-02; (c) configuration-03, and (d) configuration-04, along the *y*-axis of the simulation box. Magenta = Cl^–^ ions, black = centralized AuNP #1, AuNP #2 = green, AuNP #3 = blue, AuNP #4 = yellow and red = water.

**Figure 4:**
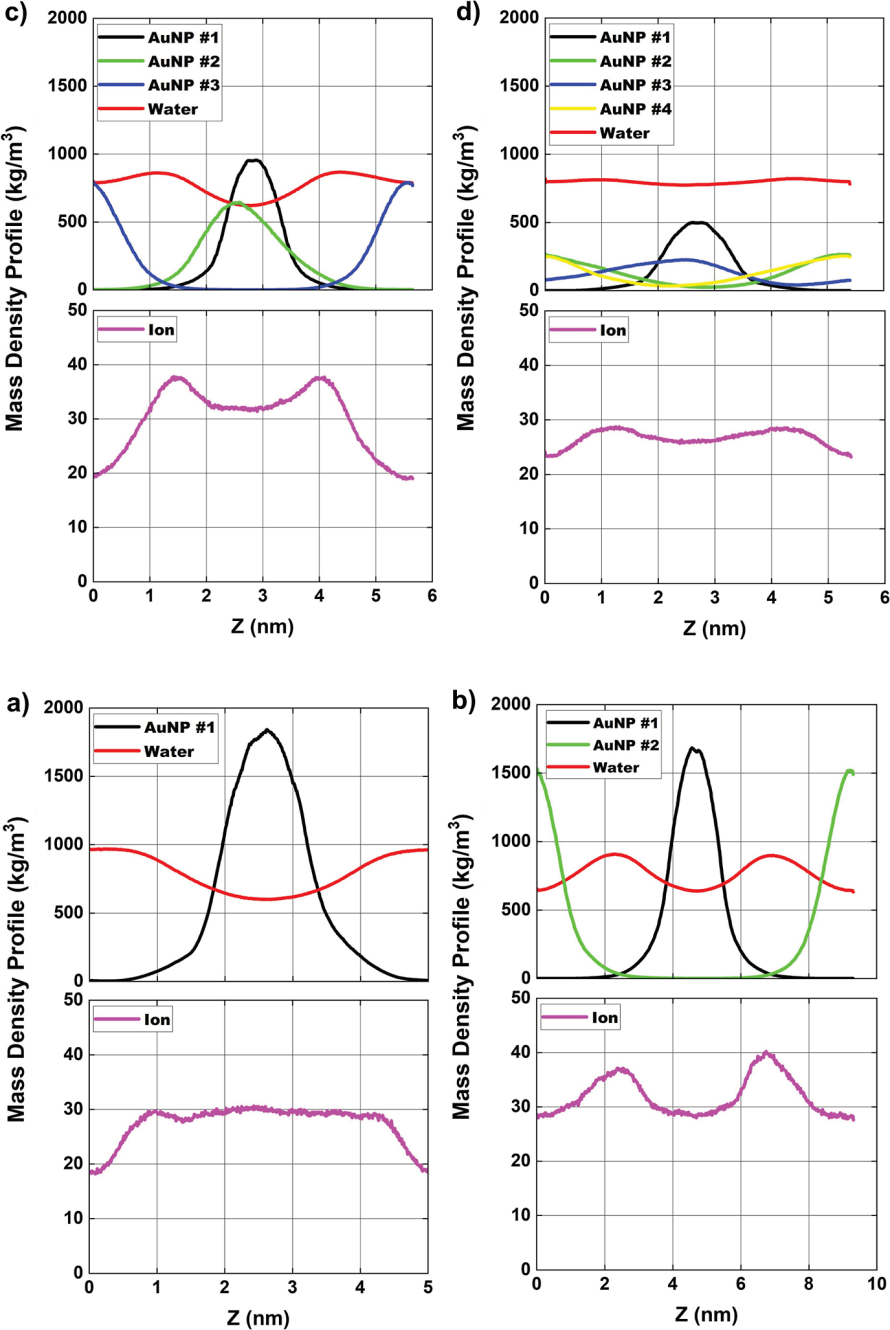
Average mass density profile [in kg/m^3^] of AuNPs and ions in solution and atomic mass for (a) configuration-01; (b) configuration-02; (c) configuration-03 and (d) configuration-04, along the *z*-axis of the simulation box. Magenta = Cl^–^ ions, black = centralized AuNP #1, AuNP #2 = green, AuNP #3 = blue, AuNP #4 = yellow and red = water.

The mass density profile of water along the *y*-axis (Figure 3) presents a behavior like that observed for the *x*-axis (Figure 2). In Figure 3a, configuration-01, the water density profile decreases in the central region. As the number of AuNPs increases (Figure 3b–d, for configurations-02, 03, and 04, respectively), water is distributed in regions between the nanoparticles, but with reduced density close to central region, reiterating that the ions are distributed mainly around the AuNPs. The mass density profile on the *z*-axis (Figure 4) confirms the observations made on the *x*- and *y*-axes, for configurations-01 and 02. For Configurations-03 and 04, the profile on the *z*-axis presents more variations in the position and width of the peaks, suggesting a less uniform vertical distribution of the AuNPs, which may indicate greater mobility in this direction. The profile on the *z*-axis presents greater dispersion and asymmetry, especially with multiple nanoparticles. The mass density profile of water molecules and chlorine ions reveals the influence of nanoparticles in the system, with local effects more visible near the particles. The results also suggest that with symmetrical and well-localized peaks of the mass density profiles we can estimate the diameter and volume occupied by AuNPs through the width at half-maximum height (fwhm) of these curves projected on each axis (presented in Table 7). The fwhm is the distance between two points on a curve where the mass density value reaches half the maximum value. The results show average diameters between 1.27 and 1.37 nm, values that suggest a volume between 1.07 nm^3^ and 1.35 nm^3^. For the volume of the AuNPs in configurations-03 and 04, we observe similar values, indicating little structural variation between AuNPs. This suggests that the presence of multiple nanoparticles may slightly influence the mass density distribution and average sizes of AuNPs. Thus, it is noted that the radial distribution function (RDF) suggests that the structural organization of AuNPs is concentration-dependent, providing guidelines for the development of controlled nanostructures, such as catalytic surfaces and electronic devices. Visual analysis of the configurations also allows a better understanding of the spatial organization between nanoparticles and ions, facilitating the interpretation of the results generated by simulations. Thus, in Figure 5, we will present a MD configuration extracted from the classical trajectory with the AuNPs in evidence. Additionally, we perform an analysis of the distance between the AuNPs via the RDFs.

**Table 7:** Average Values for the Diameter and Volume of AuNPs Considering the Average of the Values Obtained by FWHM for the Mass Density Profile of AuNPs in the *x*, *y* and *z* Directions of the Simulation Box^a^

fwhm (average, in nm)				
system	configuration-01	configuration-02	configuration-03	configuration-04
AuNP #1	1.37	1.27	1.31	1.31
AuNP #2		1.27	1.30	1.31
AuNP #3			1.30	1.30
AuNP #4				1.31
average diameter (in nm)	1.37	1.27	1.30	1.31
average volume (in nm^3^)	1.35	1.07	1.15	1.18

a AuNP volume was calculated taking into account a spherical structure for the AuNP.

**Figure 5:**
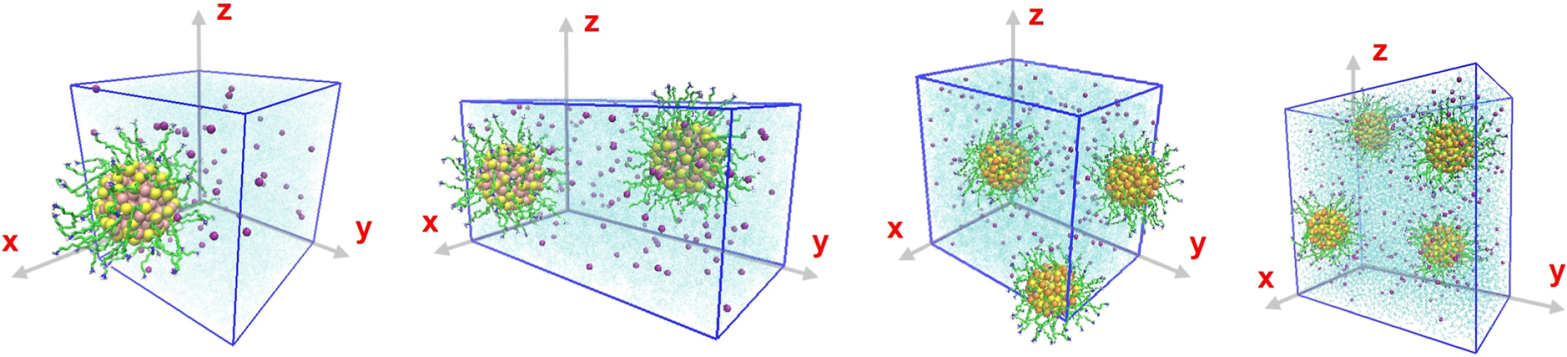
Detailed structure created by the cluster of AuNPs and ions. AuNP–Au_144_(SRNH_3_^+^)_60_. The-R-carbon chain is composed of R = C_11_H_22_ (green) and has a polar head containing an amino group (–NH_3_^+^) (red). Connected to the gold core (Au_144_) of the AuNP (yellow) through a sulfur (S) atom (orange). Configurations-1 to 4.

### 3.4 RDF

Figure 6a represents the RDF, *g*(*r*), of configuration-02 considering AuNPs #1–#2 (black line). For this system *g*(*r*) demonstrates a peak around 3.7 nm that represents the average distance between the two AuNPs in the simulation box. In Figure 6b we present the RDF for configuration-03, between AuNPs #1–#2 (black line), AuNPs #1–#3 (green line) and AuNPs #2–#3 (blue line). For these structures the RDF describes a triangular configuration whose edges are around 3.5 nm, 3.1 and 3.1 nm, respectively. In Figure 6c, we present the RDFs of configuration-04 between AuNPs #1–#2 (black line), AuNPs #1–#3 (green line), and AuNPs #1–#4 (blue line), which record peaks close to 3.7, 5.0, and 4.6 nm, respectively, for the distances between AuNPs. In Figure 6d, the analysis for configuration-04 is complemented by highlighting the RDF between AuNPs #2–#3 (black line), AuNPs #2–#4 (green line) and AuNPs #3–#4 (blue line), describing peaks located close to 4.6 nm, 5.0 and 4.7 nm, respectively. These results are systematized in Table 8. Thus, the RDFs of AuNPs revealed important information about their spatial organization in the different configurations studied. In configuration-02, a greater dispersion of AuNPs was observed, with lower average interaction intensity and greater distances between particles, indicating a less aggregated structure. Configuration-03, on the other hand, presented an opposite behavior, with greater compaction and aggregation of AuNPs, evidenced by the higher intensity of *g*(*r*) and smaller distances between AuNPs, characterizing a triangular configuration. Configuration-04 exhibited an intermediate structure, with AuNPs organized in a triangular shape with a fourth AuNP outside the plane established by the other three AuNPs. The RDFs still show a wide profile in *r*, which indicates a constant mobility of these particles, not characterizing a fixed region of these AuNPs in the simulation box.

**Figure 6:**
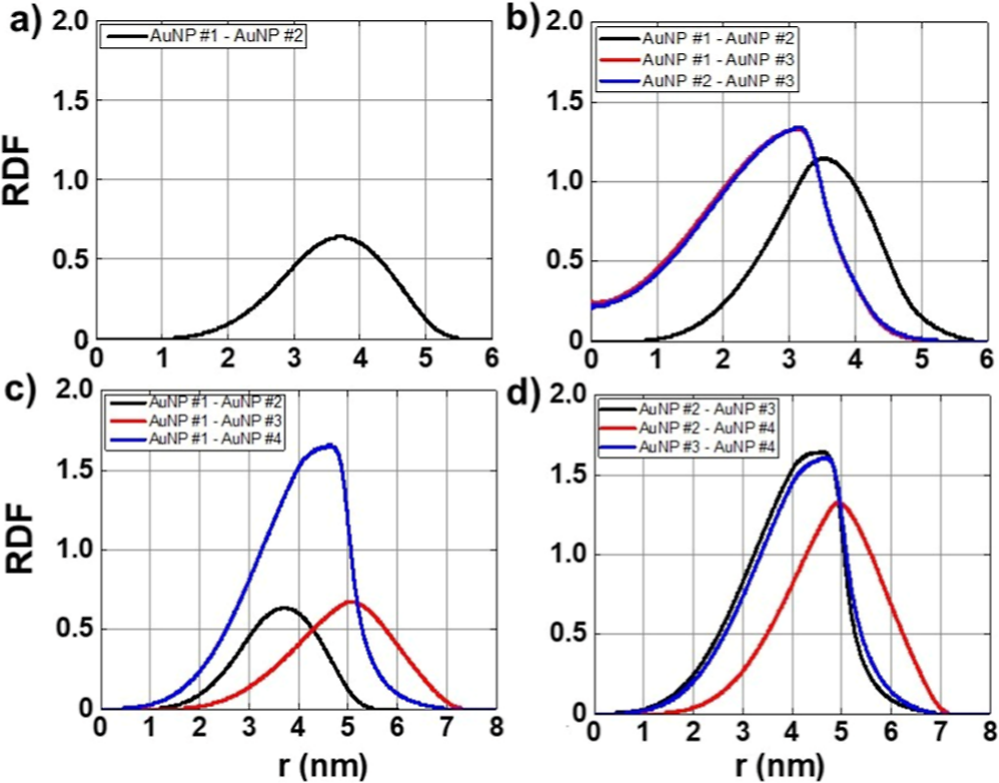
RDF obtained from MD simulations for AuNPs. (a) Configuration-02: AuNP #1–AuNP #2 (black); (b) configuration-03: AuNP #1–AuNP #2 (black), AuNP #1–AuNP #3 (green) and AuNP #2–AuNP #3 (blue); (c) configuration-04: AuNP #1–AuNP #2 (black), AuNP #1–AuNP #3 (green) and AuNP #1–AuNP #4 (blue) and (d) configuration-04: AuNP #2–AuNP #3 (black), AuNP #2–AuNP #4 (green) and AuNP #3–AuNP #4 (blue).

**Table 8:** Peak Positioning of the RDFs Indicating the Most Likely Estimated Distance between the AuNPs for Configurations 01 to 04

average distance (nm)			
system	configuration-02	configuration-03	configuration-04
AuNP #1–AuNP #2	3.7	3.5	3.7
AuNP #1–AuNP #3		3.1	5.0
AuNP #1–AuNP #4			4.6
AuNP #2–AuNP #3		3.1	4.6
AuNP #2–AuNP #4			5.0
AuNP #3–AuNP #4			4.7

### 3.5 MSD

The Einstein diffusion coefficient (MSD—Mean Square Displacement) can be used as an indicator of the mobility of AuNPs and ions in solution. Figure 7 shows the graphs related to the computational model used to calculate the mean square displacement, whose results are summarized in Table 9. When evaluating the mobility of AuNPs, we can conclude that there is a variation in the results between the simulated models, suggesting that the mobility of AuNPs can be influenced by the increase in the molar concentration of AuNPs in the solution. Taking the simulation of configuration-01 as a reference, we can see in configuration-02 that there is a variation in the mobility of AuNPs. For AuNP #1 of configuration-02, we observe a decrease of ∼14% compared to the reference (configuration-01), however, for AuNP #2 of configuration-02, there is an increase of ∼34%. On average, the mobility of AuNPs of configuration-02 increases by approximately ∼9%.

**Figure 7:**
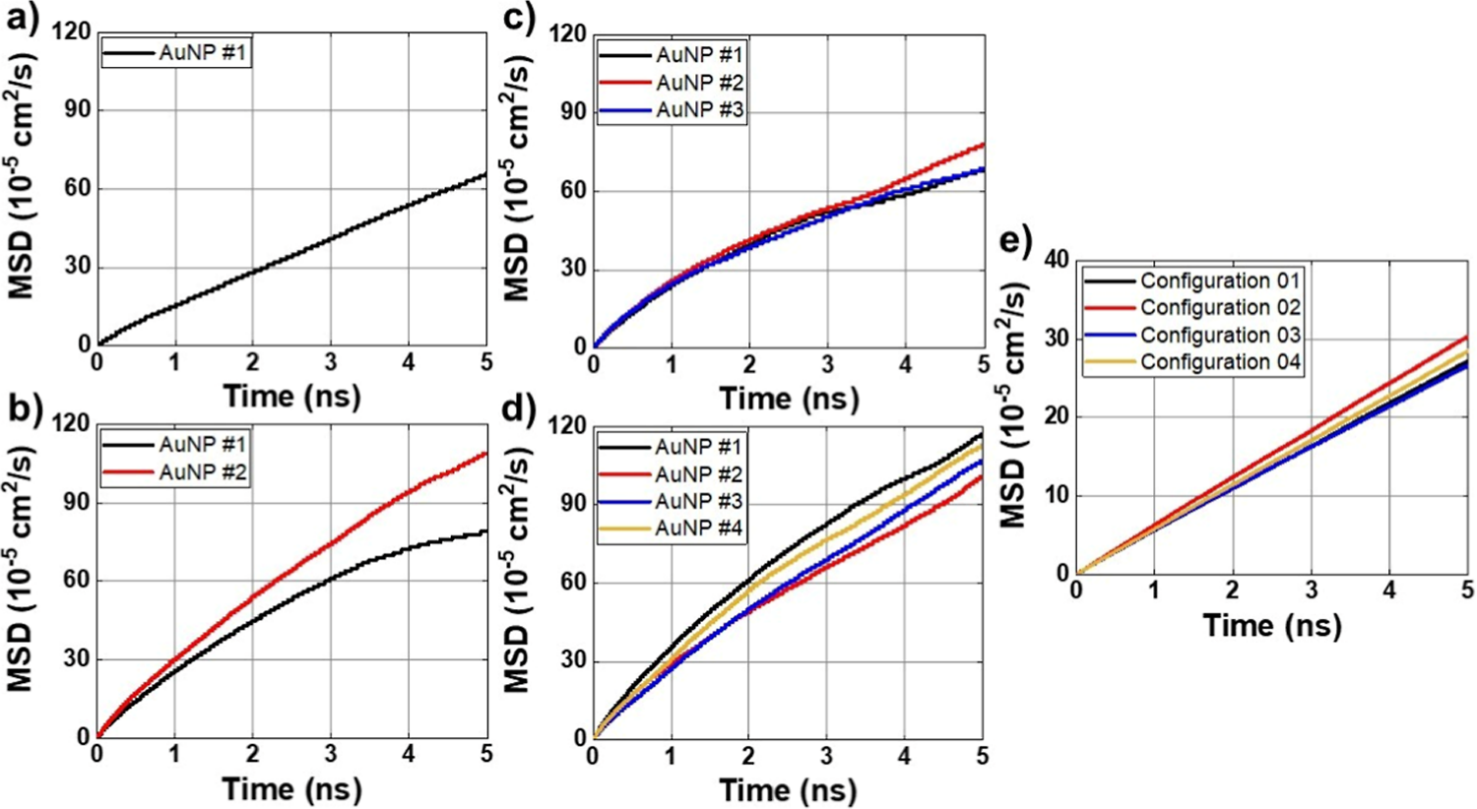
MSD curves obtained from MD simulations for AuNP. (a) Configuration-01, (b) configuration-02, (c) configuration-03, (d) configuration-04, and (e) Cl^–^ ions.

**Table 9:** Einstein Diffusion Coefficient for Each of the Nanoparticles in Configurations-01 to 04, in Water Solution, and for the Group of AuNPs^a^

MSD				
system	configuration-01	configuration-02	configuration-03	configuration-04
ions	140.59 ± 7.72	147.56 ± 5.37	130.82 ± 1.78	141.50 ± 1.01
AuNP #1	2.94 ± 0.50	2.53 ± 1.94	2.14 ± 1.91	4.52 ± 1.70
AuNP #2		3.94 ± 2.32	2.81 ± 1.40	3.71 ± 2.22
AuNP #3			2.43 ± 1.27	4.53 ± 1.36
AuNP #4				4.74 ± 0.95
AuNPs average	2.94 ± 0.50	3.23 ± 2.13	2.46 ± 1.53	4.37 ± 1.56

a Results in 10^–7^ cm^2^/s.

For configuration-03, there is a decrease in the average mobility of the AuNPs, which reflects the increase in the concentration of the AuNPs. AuNP #1 of this configuration presents a reduction of ∼27% compared to the reference; for AuNP #2 we observe a reduction of ∼4% and for AuNP #3 a reduction of ∼17%. On average, the AuNPs of this configuration are approximately ∼20% less mobile than that observed for configuration-01, in which only one AuNP is simulated. For configuration-04, which presents the lowest concentration of AuNP per simulated volume, we can observe an increase in the mobility of the AuNPs. The increases are ∼54%, 26%, 54% and 62%, respectively for each of the AuNPs compared to the reference. These results can be explained by the different interactions between the nanoparticle surface and Cl^–^ ions and the variation in the molar concentration of the AuNPs. Furthermore, when considering the interactions of the ions with the AuNPs, especially the properties such as the affinity of the ions for the surface, influenced by factors such as interaction energy (*E*_C_ e *E*_LJ_), also play a significant role in the observed mobility. In the context of this study, the configurations with the highest ion mobility were configuration-02 and configuration-04 and represent a higher degree of freedom of the AuNPs. Despite this mobility, previous results show that there is a preferential region of relative positioning of these AuNPs.

## 4 Conclusion

In this study, we conducted MD computational simulations to investigate the energetic, structural properties and dynamics of interactions of functionalized AuNPs, Au_144_(SRNH_3_^+^)_60_, in aqueous solution containing chlorine ions (Cl^–^) at different concentrations. The results, obtained from thermodynamically equilibrated and converged MD trajectories, indicate that the Coulomb interaction energy between AuNPs and chlorine ions showed that the AuNP–AuNP self-interaction is quite high in all configurations and that higher molar concentrations of AuNPs in solution influence the interactions with water molecules and chlorine ions in the solution, resulting in a reduction of the interactions between AuNPs and water and an increase in the interactions with ions. For the average number of HBs formed between the nanoparticles and water molecules we obtain quite stable results, which can be considered converged, regardless of the concentration studied, suggesting that the increase in concentration does not drastically alter the dynamics of these interactions. Previous results have shown that the interactions between AuNPs and water are energetically favorable and strong, driven mainly by electrical interactions. The high hydration of the nanoparticles in the terminal regions of the functionalization, evidenced by the high number of HBs, confirms the dynamic results for HBs (the lifetime and the Gibbs free energy for rupture of an HB). This reinforces the potential of AuNPs in applications that depend on robust and energetically stable aqueous colloidal systems, such as stable AuNP dispersions for drug delivery systems, biosensors and catalysis. Our results highlight the key role of the functionalized region in mediating the interaction of AuNPs with the ionic environment, particularly through strong NH_3_^+^–Cl^–^ electrostatic interactions and a stable hydrogen-bonding network between AuNP and water molecules that supports colloidal stability. The clear distinction between the inert gold core and the responsive ligand shell reinforces the importance of surface design in tailoring AuNPs for biomedical, sensing, and catalytic applications.

The spatial distribution of nanoparticles and ions in solution shows that AuNPs significantly influence the organization of water and ions in their vicinity. Chlorine ions concentrate preferentially in regions between AuNPs, highlighting the competition of these particles in filling the AuNP solvation shell. The structural analysis based on mass density profiles allowed estimating the diameter of AuNPs and facilitates the understanding of the interaction between the components of the system. The average values show that AuNPs have an average diameter of ∼1.31 nm, resulting in an average volume close to ∼1.18 nm^3^. The distances between AuNPs were also estimated and depend on the concentration of these particles in solution. In more complex configurations, distances between AuNPs can reach up to ∼4 nm with high mobility of AuNPs, as could be observed with the results obtained for MSD.

Overall, this study demonstrates how the charge and concentration of AuNPs influence their interaction with the surrounding medium, enabling adjustments in surface functionalization, ionic composition, and nanoparticle density for specific applications. Future studies may expand upon these findings by incorporating different ionic environments and functional groups, enhancing control over the behavior of AuNPs in complex systems.

## Supplementary Material



## Notes

Figure S1—RMSD graphs for Configuration-01: AuNP, ions, water molecules; Figure S2—RMSD graphs for Configuration-02: AuNP, ions, water molecules; Figure S3—RMSD graphs for Configuration-03: AuNP, ions, water molecules; Figure S4—RMSD graphs for Configuration-04: AuNP, ions, water molecules (PDF)
