# Supplementary material for: Investigating the Impact of Positively Charged Gold Nanoparticle (AuNP) Concentration in Water/Cl– Solutions Using Molecular Dynamics Simulations
Source: ACS Omega. 2025 May 14;10(20):20610–22. doi: 10.1021/acsomega.5c01441 (PMC12120595; doi:10.1021/acsomega.5c01441)
Supplement: Supplementary file 1 [file ao5c01441_si_001.pdf]

SUPPORT MATERIAL

Investigating the impact of positively charged gold nanoparticle (AuNP) concentration in water/Cl^- solutions using molecular dynamics simulations

Esequias Coelho[a], Douglas Xavier de Andrade[b], Agnaldo Rosa de Almeida[c] and Guilherme Colherinhas[a*]

[a] *Instituto de Física, Universidade Federal de Goiás, 74690-900, Goiânia, GO, Brazil.*
[b] *Instituto Federal de Educação, Ciência e Tecnologia de Goiás, 74968-755, Aparecida de Goiânia, GO, Brazil.*
[c] *Campus Anápolis de Ciências Exatas e Tecnológicas, Universidade Estadual de Goiás, 75.132-400, Anápolis, GO, Brazil.*
** corresponding author: gcolherinhas@ufg.br*

Abstract: This study presents a detailed analysis of the interactions between positively charged gold nanoparticles $Au_{144}(SRNH_3^+)_{60}$ and chloride ions (Cl^-) in aqueous solution, using molecular dynamics simulations. Four systems with varying amounts of chloride ions were investigated: 60 Cl^-, 120 Cl^-, 180 Cl^-, and 240 Cl^-, alongside varying quantities of nanoparticles. The focus of this research is to elucidate the energies involved, hydrogen bonding patterns, and radial distribution of ions around the gold nanoparticles, providing a fundamental basis for evaluating the potential applications of these systems in disease treatment. The results reveal significant differences in the Coulomb and van der Waals interaction energies between nanoparticles and ions, as well as between nanoparticles and water molecules. Furthermore, this study highlights the patterns and lifetimes of hydrogen bonds between nanoparticles and water molecules, along with the mobility of system components in solution. These findings have important implications for potential applications in bio-nanotechnology, offering a deeper understanding of the interactions between ions and gold-based nanoparticles.

Keywords: *Molecular Dynamics; Gold Nanoparticle; Ionic Interaction; H-Bond; H-Bond Lifetime.*

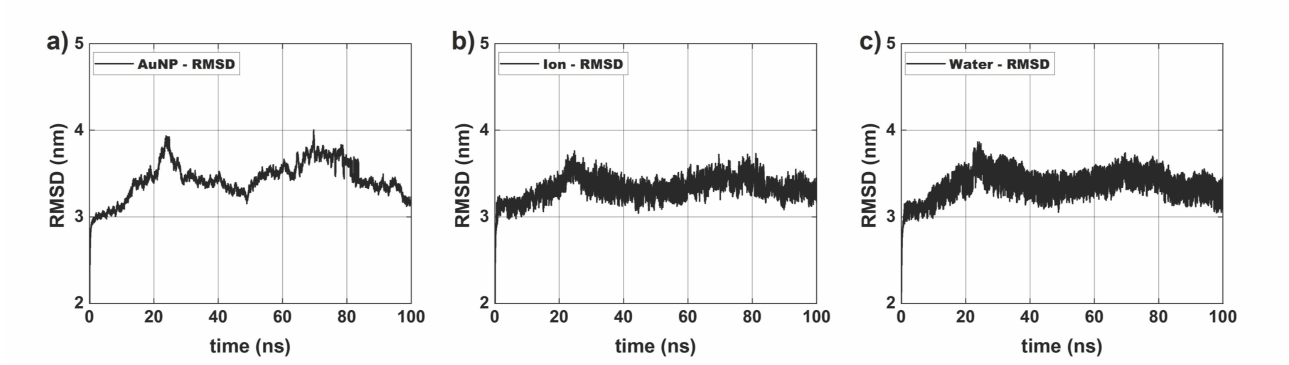

Figure S1. *RMSD graphs for Configuration-01. (a) AuNP; (b) Ions; and (c) Water molecules.*

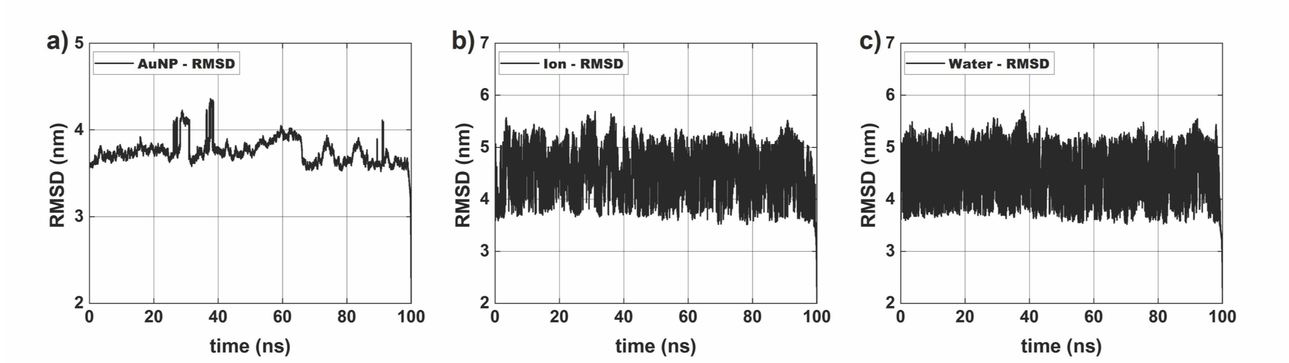

Figure S2. *RMSD graphs for Configuration-02. (a) AuNP; (b) Ions; and (c) Water molecules.*

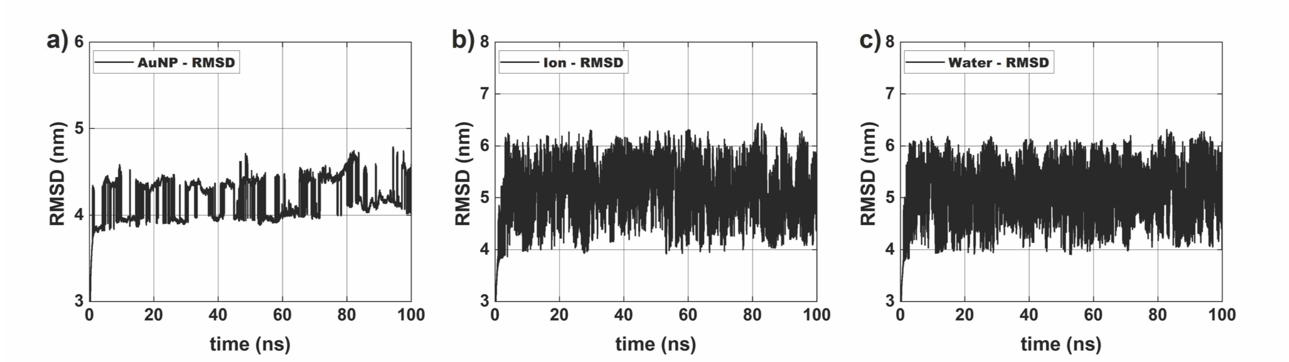

Figure S3. *RMSD graphs for Configuration-03. (a) AuNP; (b) Ions; and (c) Water molecules.*

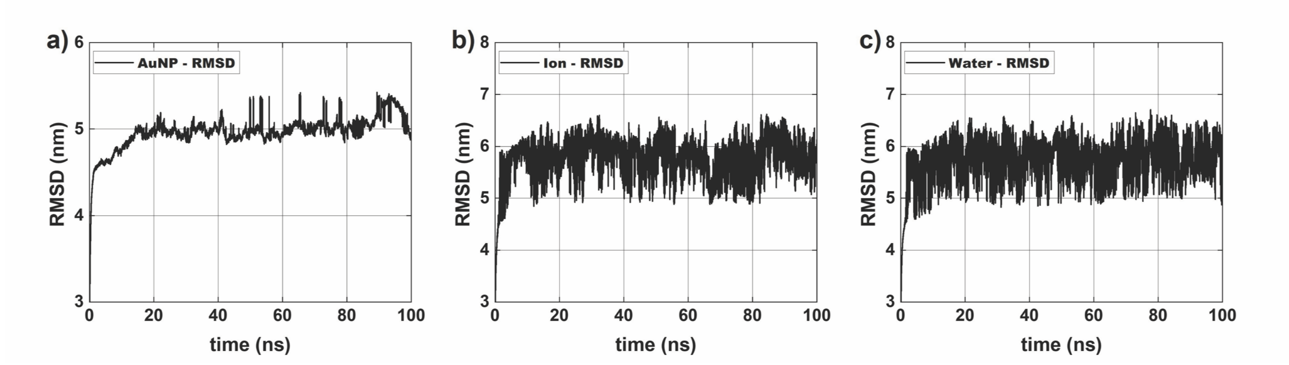

Figure S4. *RMSD graphs for Configuration-04. (a) AuNP; (b) Ions; and (c) Water molecules.*
